# Multi‐omic analysis in normal colon organoids highlights *MSH4* as a novel marker of defective mismatch repair in Lynch syndrome and microsatellite instability

**DOI:** 10.1002/cam4.6048

**Published:** 2023-05-10

**Authors:** Matthew Devall, Mourad W. Ali, Stephen Eaton, Daniel J. Weisenberger, Matthew J. Reilley, Steven M. Powell, Li Li, Graham Casey

**Affiliations:** ^1^ Center for Public Health Genomics University of Virginia Charlottesville Virginia USA; ^2^ Department of Family Medicine University of Virginia Charlottesville Virginia USA; ^3^ Department of Biochemistry and Molecular Medicine University of Southern California Los Angeles California USA; ^4^ University of Virginia Comprehensive Cancer Center, University of Virginia Charlottesville Virginia USA; ^5^ Digestive Health Center University of Virginia Charlottesville Virginia USA; ^6^ Department of Public Health Sciences University of Virginia Charlottesville Virginia USA

**Keywords:** colon organoids, colorectal cancer, Lynch syndrome, microsatellite instability

## Abstract

**Introduction:**

Lynch syndrome (LS) is a hereditary condition that increases the risk of colorectal (CRC) and extracolonic cancers that exhibit microsatellite instability (MSI‐H). MSI‐H is driven by defective mismatch repair (dMMR), and approximately 15% of nonhereditary CRCs also exhibit MSI‐H. Here, we aimed to better define mechanisms underlying tumor initiation in LS and MSI‐H cancers through multi‐omic analyses of LS normal colon organoids and MSI‐H tumors.

**Methods:**

Right (*n* = 35) and left (*n* = 23) colon organoids generated from normal colon biopsies at routine colonoscopy of LS and healthy individuals were subjected to Illumina EPIC array. Differentially methylated region (DMR) analysis was performed by DMRcate. RNA‐sequencing (*n* = 16) and bisulfite‐sequencing (*n* = 15) were performed on a subset of right colon organoids. CRISPR‐cas9‐mediated editing of MMR genes in colon organoids of healthy individuals was followed by quantitative PCR of *MSH4*. The relationship between *MSH4* expression and tumor mutational burden was further explored in three independent tumor data sets.

**Results:**

We identified a hypermethylated region of *MSH4* in both the right and left colon organoids of LS versus healthy controls, which we validated using bisulfite‐sequencing. DMR analysis in three gastrointestinal and one endometrial data set revealed that this region was also hypermethylated in MSI‐H versus microsatellite stable (MSS) tumors. *MSH4* expression was increased in colon organoids of LS versus healthy subjects and in publicly available MSI‐H versus MSS tumors across four RNA‐seq and four microarray data sets. CRISPR‐cas9 editing of *MLH1* and *MSH2*, but not *MSH6*, in normal colon organoids significantly increased *MSH4* expression. *MSH4* expression was significantly associated with tumor mutational burden in three publicly available data sets.

**Conclusions:**

Our findings implicate DNA methylation and gene expression differences of *MSH4* as a marker of dMMR and as a potential novel biomarker of LS. Our study of LS colon organoids supports the hypothesis that dMMR exists in the colons of LS subjects prior to CRC.

## 1 INTRODUCTION

Approximately 15% of colorectal cancers (CRCs) display high levels of microsatellite instability (MSI‐H), while the remainder can be broadly deemed as being microsatellite stable (MSS). In CRC, the vast majority (~80%) of tumors are derived from nonhereditary mechanisms. Primarily, these tumors evolve mostly as a result of DNA hypermethylation and inactivation of the mismatch repair (MMR) gene: MutL homolog 1 (*MLH1*).^1^, ^2^ However, individuals with Lynch syndrome (LS) harbor mutations that lead to a hereditary predisposition to some cancers, including CRC. LS is an autosomal dominant disorder resulting from inherited mutations in MMR genes: *MLH1*, MutS homolog 2 (*MSH2*), *MSH6* or PMS1 homolog 2, mismatch repair system component (*PMS2*), or through a rare deletion of the *Epithelial cell adhesion molecule* (*EPCAM*), which inactivates *MSH2*.^3^ Mutations in *MSH2* and *MLH1* are responsible for approximately 70% of LS.^4^ Inherited mutations in these genes lead to an increased risk of colonic and extracolonic (primarily endometrium and stomach) tumor development.^5^ Individuals with LS often present with earlier onset cancers. Differences in lifetime risk estimates for LS subjects are thought to be driven by disease variant pathogenicity, the specific MMR gene involved, and other factors.^6^, ^7^, ^8^ One challenge for the clinical management of LS is the need to identify additional molecular markers driving risk. Such markers have the potential to improve screening and provide insight into the early development of cancer.

The MMR system is a highly conserved, postreplicative process involved in maintaining the fidelity of genetic information passed from templates to daughter strands.^9^ This process involves a staged, coordinated effort from various MMR proteins. First, surveillance heterodimers MutSα and MutSβ aim to identify specific mismatches. MutSα (MSH2‐MSH6) primarily recognizes single‐base mismatches or 1–2 nucleotide insertion/deletion loops (IDLs),^9^ whereas MutSβ (MSH2–MSH3) has a higher recognition affinity for larger IDLs.^10^ Following identification, the appropriate heterodimer will bind to the target site and recruit MutLα (MLH1‐ PMS2)^10^ in a process involving Exonuclease 1 (EXO1).^11^ Subsequently, DNA polymerases and replication factors augment re‐synthesis at the target sites. Deficient MMR (dMMR) leads to a failure to correct replication errors at microsatellite repeats^12^ and results in tumors that exhibit the hypermutator phenotype: MSI‐H. Individuals with LS exhibit an increased risk of developing CRC with MSI‐H given that they already harbor germline or de novo mutations in one copy of a specific MMR gene. Following a somatic mutation of the normal copy of that MMR gene (“second‐hit hypothesis”^13^), the resulting dMMR affects the cell's ability to repair DNA and results in increased mutational burden.^14^

Aberrant levels of DNA methylation are a hallmark of CRC. However, most research into LS has focused on the identification of novel, inherited gene variants. There has been comparatively little research in LS subjects into the molecular mechanisms underlying tumor development that help to drive the dMMR/MSI‐H tumor phenotype. Although some studies have attempted to address the role of DNA methylation in LS, these studies have primarily been focused on blood.^15^ Given the tissue and cell‐specific nature of DNA methylation, blood analyses may not provide appropriate insight into CRC disease pathology, which likely originates from the stem‐cell compartment of the colon crypt. In addition, few studies have considered defining the relationship between LS and nonhereditary MSI‐H.

In this study, we hypothesized that a multi‐omic, comparative analysis of organoids generated from normal colons of LS versus healthy subjects would provide insight into epigenetic and transcriptomic differences occurring in the high‐risk, LS population. Colon organoids are an ideal model system in which to study this as they are comprised predominantly of epithelial stem‐cell niche cells, which have been hypothesized to be the origin for CRC.^7^, ^8^ We further hypothesized that differences observed in LS organoids may be extended to non‐hereditary MSI‐H tumors. Our study led to the identification of the meiosis‐associated gene *MSH4*, an MMR gene not previously implicated in LS, as a potential novel marker of dMMR/MSI‐H/LS.

## 2 MATERIALS AND METHODS

### 2.1 Subject recruitment and exclusion criteria

Subjects scheduled for screening or surveillance colonoscopies were enrolled after providing informed consent under an approved Institutional Review Board protocol at the University of Virginia (IRB‐HSR #19710). Subjects were recruited between July 2017 and March 2019 and agreed to donate biopsies from both the right and left colon. Healthy control subjects were excluded from this study if they had a personal or family history of CRC or a personal history of inflammatory bowel disease. All procedures were performed in accordance with relevant guidelines and regulations and were consistent with those required by both the National Institutes of Health and the University of Virginia. Written informed consent has been obtained from the patient(s) to publish this paper and the study was conducted in accordance with U.S. Common Rule.

### 2.2 Establishment and passaging of colon organoids

Colon organoids were developed from biopsies of either right or left colon using a modification of the method described by Sato et al. (2011).^7^, ^16^ Biopsies were obtained immediately distal to the hepatic flexure (right colon) or immediately distal to the splenic flexure (left colon) using standard forceps at screening or routine colonoscopy. Whole crypts were isolated by gentle mechanical disruption and embedded in Matrigel.^7^ Growth media included advanced DMEM/F12 (Gibco #12634‐010), 1X Pen‐Strep (Gibco #15140‐122: (100 U/mL penicillin, 100 μg/mL streptomycin)), 10 mM Hepes (Gibco #15630–080), 1x N2 Supplement (Gibco #17502–048), 1x B27 Supplement (Gibco #17504–44), 1x GlutaMAX (Gibco #35050–061), 1 mM N‐acetylcysteine (Sigma #A9165), 10 nM gastrin (Sigma #G9020), 50% L‐WRN conditioned media, 500 nM A83‐01 (R&D #2939), 10uM SB202190 (R&D #1264), 10 mM nicotinamide (Sigma #N6636), 50 ng/mL EGF (Life Technologies #PHG0311L), and 10 μM SB202190 (R&D #1264). Colon organoids were fed with fresh growth media every 3 days and passaged as needed (typically every 3–5 days) in 48‐well culture plates, as previously described.^16^ Briefly, organoids were gently dissociated through scratching of the Matrigel using a 1000 μL pipette tip. They were then suspended in a 500 μL wash medium advanced DMEM/F12 (Gibco #12634–010), 1X Pen‐Strep (Gibco #15140–122: (100 U/mL penicillin, 100 μg/mL streptomycin)) and transferred to an Eppendorf tube. Here, the suspension was centrifuged at 300 × *g* for 5 min (4°C). Following this, the majority of supernatant was removed and the organoid pellet was resuspended in 1 mL TrypLE Express (Gibco, #12,604,013) and incubated at 37°C for a period of 10 min. After the incubation period expired, a 2 mL wash medium was added. Organoids were dispersed in this wash medium using a 25G needle and 1 mL syringe by slowly passing them through the needle between six and eight times. The mixture containing these organoids was then centrifuged a second time at 300 × *g* for 5 min (4°C). Eppendorfs were removed from the centrifuge and supernatant was carefully removed from the pellet containing organoids. This pellet was resuspended in an appropriate volume of Matrigel to seed 30 μL per well at a 1:3 split ratio. Plates were incubated at 37°C for 15 min to polymerize Matrigel and then 500 μL growth media was added. For organoid harvesting, 300 μL of Buffer RA1 and 6 μL of TCEP was added to each pellet (NucleoSpin RNA XS Kit (Takara Bio: 740990.250)).

### 2.3 DNA extraction of colon organoids

Genomic DNA was extracted from colon organoids using the Qiagen UCP DNA kit (Catalog No: 56204; Qiagen; Hilden, Germany), with a few exceptions. For elution, a 5 min final incubation of Buffer AUE was preferred to increase yield. Further, the elution step was carried out twice using two volumes of 25 μL. DNA quality was assessed using gel electrophoresis to confirm that DNA was not heavily degraded.

### 2.4 RNA Extraction and sequencing of colon organoids

Total RNA was extracted using NucleoSpin RNA XS Kit (Takara Bio: 740990.250). All samples used for library preparation had RNA integrity numbers above 9.8, as measured by Agilent 4200 Tapestation (G2991BA). Library preparation and RNA‐seq were carried out according to Illumina protocols following ribosomal depletion at the Northwest Genomics Center of the University of Washington. Paired‐end, 100 bp sequencing was performed using the Illumina NovaSeq 6000. An average of 51.98 million reads were uniquely mapped to the GrCh38 for each sample, with an efficiency of 67.78% using STAR and RSEM.^17^, ^18^

### 2.5 Preprocessing of DNA methylation microarray data in colon organoids

Bisulfite‐converted DNA quantity and bisulfite conversion completeness were assessed for each sample using a panel of MethyLight‐based real‐time PCR quality control assays, as described previously.^19^ DNA methylation data were generated using the Illumina Infinium MethylationEPIC Kit (herein EPIC array; Catalog No: 20042130; Illumina; San Diego, California, USA) for right (*n* = 35) and left (*n* = 23) colon organoids generated from LS and control individuals at the USC Norris Molecular Genomics Core Facility. For right colon organoids, data were generated in two independent batches and analyzed together. Stratified quantile normalization was preferred as the normalization method for case–control differences. The method was used under default settings while considering sample gender.^20^ For this analysis, Sentrix chip and sample positions were used as adjustment factors^21^ to account for technical variation prior to the analysis of differentially methylated regions (DMRs).^22^

### 2.6 General steps for processing EPIC array data across cohorts

Samples were excluded if they did not pass sex or ethnicity checks performed in SeSAMe.^23^ Samples were processed in minfi using a detection P value of <0.01 for probe removal.^24^ Probes were also removed if (1) the probes contained an SNP at the CpG or at the single base extension or (2) were cross‐hybridizing, were on either sex chromosome, or (3) were in a non‐CpG context. Further, a parallel beta processing of probes was performed using SeSAMe.^23^ All additional probes that failed in at least one sample were removed prior to the fitting of the model. Beta values were adjusted for technical variation using the COMBAT function in ChAMP^21^ prior to DMR analysis in DMRcate.^22^ DMR annotation was also performed using *DMRcate*. Overlapping bed regions across different analyses were determined through the use of the R package, *bedR*.^25^

### 2.7 Analysis of publicly available DNA methylation data sets

We downloaded and processed data from The Cancer Genome Atlas (TCGA) ‐colon adenocarcinoma (‐COAD) and ‐stomach adenocarcinoma (‐STAD) cohorts^26^, ^27^, ^28^ and ‐Uterine Corpus Endometrial Carcinoma (‐UCEC). Raw IDAT files were downloaded from TCGAbiolinks.^29^ Consensus purity estimates (CPE)^30^ and mDNAsi scores^31^ were downloaded for each sample. Numerous purity measures were missing for STAD, so this covariate was excluded. Functional normalization, which was designed for the analysis of cancer data sets, was applied using *minfi* ^24^ while specifying a gender. Principal component analysis was performed to identify initial outliers in technical replicates. In their absence, the replicate with the lowest mean DNA methylation level was excluded. Sentrix chips were excluded if they contained only data from either MSI‐H or MSS/MSI‐Low (MSS/MSI‐L) tumors.

### 2.8 Sample selection and preprocessing of external, publicly available DNA methylation datasets

#### 2.8.1 TCGA‐COAD

For the analysis of TCGA‐COAD DNA methylation data, a total of 11 Asian samples were removed as they were deemed too small a subset to reduce heterogeneity. Samples were also excluded if colon location data were absent. A total of 149 samples were included in this analysis, with 36 originating from MSI‐H tumors. Sentrix chip and position were used as adjustment covariates for the COMBAT function of the ChAMP package.^21^

#### 2.8.2 TCGA‐STAD

Samples without race, MSI status, mDNAsi, stage, age, or gender information were excluded. Samples were then checked for MSI status representation across each Sentrix chip. A total of 202 samples were considered for analysis.

#### 2.8.3 TCGA‐UCEC

A total of 295 tumors were considered for inclusion. Samples were removed if they were (1) not of endometrial origin, (2) not classified as “endometrioid adenocarcinoma” or “serous cystadenocarcinoma.” or (3) missing data for CPE, race, mDNAsi, MSI status, age, or tumor stage. Samples were then analyzed for adequate representation of MSI status across Sentrix IDs.

#### 2.8.4 GSE68060

Raw IDAT files containing red and green probe intensities were downloaded from gene expression omnibus (GEO),^32^ accession GEO68060. Data were processed in a manner similar to TCGA cancer cohorts. To limit the effects of genetic ancestry on baseline DNA methylation levels, our analysis was limited to the study of Spanish patients. Samples were removed if they (1) presented as large outliers on PCA, (2) had missing information on MSI status, (3) failed sex checks, or (4) were placed on a Sentrix chip that contained only MSI‐H or MSS/MSI‐L data. This limited the final study population to 23 samples. As previously, beta values were adjusted for Sentrix chip prior to DMR analysis.

### 2.9 Regression models used for DMR identification

*DMRcate* ^22^ was used under default settings, except that lambda = 1000, C = 4, and the minimum number of probes required for a DMR was set to 7. C was set a 2 for HM450 analysis. An absolute mean beta value shift of 5% was required for DMR reporting. Regression models were fitted for beta values for (1) right colon organoid, (2) left colon organoid, (3) TCGA‐COAD, (4) TCGA‐STAD, (5) TCGA‐UCEC, and (6) GSE68060.

1. DNAm ~ Age + Gender + Batch + Previous Smoked + LS
2. DNAm ~ Age + Gender + Previous Smoked + LS
3. DNAm ~ Age + CPE + Ethnicity + Tissue + Stage + Gender + mDNAsi + MSI
4. DNAm ~ Age + Ethnicity + Stage + Gender + mDNAsi + MSI
5. DNAm ~ Age + CPE + Ethnicity + Stage + mDNAsi + MSI
6. DNAm ~ MSI

### 2.10 Bisulfite sequencing and data processing of the *MSH4* locus

Paired‐end reads were sequenced for each sample across the *MSH4* locus (chr1:76262302–76,262,889; 587 bp). Given the length of the region, three overlapping libraries were constructed. For each, a methylated region of DNA obtained after treatment with the *M.Sss*I methylase was also sequenced, and this served as a positive control for adequate bisulfite treatment at each site. Reads were analyzed using *trim_galore*.^33^ Reads were trimmed with a quality score of less than 30; 6 bp of the 3′ ends of both reads R1 and R2 were also trimmed. Samples were aligned to an in silico bisulfite‐treated version of hg19 through *Bismark* ^34^ with the following parameters: ‐‐score_min L,0,‐0.4 ‐‐non_directional. DNA methylation values were then extracted using Bismark. Data were then imported into *methylKit* for regression analysis.^35^ In the case of overlapping sites across libraries, the library with the highest DNA methylation level in the *M.Sss*I‐treated sample at that site was considered. Differential DNA methylation was calculated using the chi‐squared test in *methylKit*. Bisulfite conversion appeared to fail for one sample, which was removed prior to analysis.

### 2.11 RNA‐sequencing analysis of publicly available cohorts

Three TCGA data sets (COAD, STAD, and UCEC) were selected for RNA‐sequencing analysis (RNA‐seq) to measure differences in expression between MSI‐H and MSS/MSI‐L tumors. For TCGA‐COAD, data analysis was performed on 294 samples, as previously described.^36^ Unless otherwise stated, samples were excluded if they belonged to a duplicate already used within the study or if MSI information was missing. Cancer stages were broadly categorized into main hierarchical groupings: Stage I–V, subclasses, that is, Stage IIA, were grouped into the main class (Stage II). For TCGA, raw HT‐Seq counts were downloaded from the R package *TCGAbiolinks*.^29^ As with our previous study in TCGA‐COAD, MSI‐low, and MSS samples were grouped together for comparison to MSI‐H.

#### 2.11.1 TCGA‐UCEC

Samples were excluded from TCGA‐UCEC if they were not diagnosed with either endometrial adenocarcinoma or serous cystadenocarcinoma, they were not resected from endometrium, they did not have existing consensus purity estimates or sample age or race were not reported. A total of 440 samples were used in this analysis. Cell composition estimated for six‐cell populations (ciliated and unciliated epithelial cells, fibroblasts, endothelial cells, lymphocytes, and macrophages) were calculated using CIBERSORTx^37^ using single‐cell data derived from a previously published data set.^38^

#### 2.11.2 TCGA‐STAD

A total of 342 samples were included in the final regression. Single‐cell deconvolution was performed using single‐cell data generated by Zhang et al.^39^ Cell scores were generated for T, epithelial, mast, macrophage; B, endothelial, fibroblast, endocrine, and parietal cells using a single‐cell data matrix consisting of 8563 cells.

### 2.12 Cell composition analysis

Raw counts were downloaded from TCGAbiolinks.^29^ For TCGA‐COAD, data analysis was performed as previously described.^36^ Single‐cell deconvolution performance was determined by CIBERSORTx using transcripts‐per‐million values,^37^ and second in‐house, through manual inspection of the correlation of gene expression markers to cell scores. TCGA‐UCEC was processed under largely default parameters, except for the following: minimum expression = 0.6; barcode gene range = 100–600. Similarly, TCGA‐STAD data had the following exceptions: minimum expression = 0.7; barcode gene range = 300–500. For both studies, Q values were set to 0.01, S‐mode batch correction was applied, cell scores were quantified in absolute mode and 500 permutations were used for cell score quantification.

### 2.13 Regression analysis: RNA‐seq data

Raw counts were imported into DESeq2 and the optimized FDR thresholding approach was used to determine gene significance.^40^ Cell composition scores were used to adjust for the effects of cellular heterogeneity on the differentially expressed gene (DEG) reporting for TCGA‐UCEC and TCGA‐STAD data.^40^ This method was previously used for our analysis of TCGA‐COAD data.^36^ For LS colon organoids, gene counts were generated for each sample and analyzed in DESeq2.^40^ A summary of the covariates used in each regression was as follows:

1. ExprUCEC ~ Purity + Age + Ethnicity + Stage + Cell Scores + Status
2. ExprSTAD ~ Age + Sex + Stage + Cell Scores + Status
3. ExprLS ~ Age + Previous Smoked + LS

### 2.14 Microarray analysis of publicly available data

For microarray studies, data were processed in a manner dependent upon the array manufacturer. Data were downloaded either from Gene Expression Omnibus^32^ or ArrayExpress.^41^ For E‐MTAB‐8148^42^ (Illumina), probes were removed if they had a detection P value of <0.05 in at least one sample. Background correction and quantile normalization were then carried out in *limma* ^43^ using negative control probes for correction and both negative and positive controls for normalization. Affymetrix array data were downloaded from E‐GEOD‐41258,^44^ E‐GEOD‐26682,^45^ and GSE13294.^46^ E‐GEOD‐26682^45^ contained data generated on two different array platforms. As such, independent regressions were performed for each subset. For each study, PCA was performed to identify potential outliers. Samples were also removed following a visual inspection of median probe intensities; if the array did not contain the representation of both MSI‐H and MSS tumors or if they were missing relevant covariates that were used in the regression of each study. Probes that mapped to more than one unique identifier were removed from downstream analysis. Probes were also filtered based on a study‐specific minimum cutoff for intensity levels. A total of 140 and 159 samples survived preprocessing in “batch33” and “batch44” for E‐GEOD‐26682,^45^ respectively. A total of 211, 147, and 155 were used for E‐MTAB‐8148,^42^ E‐GEOD‐41258^44^ and GSE13294,^46^ respectively.

### 2.15 Microarray regression analysis of publicly available data

Linear models were employed for regression analysis for each microarray data set was performed for each data set independently in limma^43^ on log‐transformed expression values. A summary of the covariates used in each regression is as follows:

1. ExprEMTAB8148 ~ sentrixID + Age + Gender + Location + Status
2. ExprEGEOD26882 ~ sentrixID + Age + Status
3. ExprEGEOD41258 ~ Stage + Age + Gender + Location + Status
4. ExprGSE13294 ~ Age + Gender + Status

The R package *metaSeq* ^47^ was used for meta‐analysis of microarray data using the other.oneside.pvalues() function. Genes were not filtered based on p‐value prior to meta‐analysis. Instead, genes were only considered if they displayed concordant directions of effect across each cohort. Independent weights were assigned as continuous variables which corresponded to the study size of each cohort included in the meta‐analysis.

### 2.16 Survival analysis of TCGA data sets

We downloaded data on overall survival (OS) from cBioPortal^48^ for TCGA‐COAD, TCGA‐STAD, and TCGA‐UCEC. Cox proportional hazards models were fitted to test for the association between *MSH4* expression (tertile: low, medium, high) and OS using the coxph() function of the survival package (version 3.5–5). To account for potential differences between survival metrics and factors such as MSI status,^49^ we adjusted for study‐specific covariates:

1. COAD ~ Age + Gender + MSI status + Stage + MSH4
2. STAD ~ Age + Gender + MSI status + Stage + MSH4
3. UCEC ~ Age + MSI status + Stage + MSH4

### 2.17 CRISPR‐Cas9 editing of MMR genes and analysis of effects on target gene and *MSH4* expression

Clustered regularly interspaced short palindromic repeats (CRISPR) guide RNAs (gRNAs) targeting candidate genes (at least two gRNAs per gene) were purchased from Synthego (Menlo Park, CA) guide sequences; *MSH2* gRNA1: 5′ UCA AAC UGA GAG AGA UUG CC 3′, gRNA2: 5′ GUU AAA AUG UCC GCA GUU GA 3′, gRNA3: 5′ GAU UCC AUA CAG AGG AAA CU 3′ (deletion size: ~0.135 kb); MSH6; gRNA1: 5′ AAC AGU UGU GAC UUC UCA CC 3′, gRNA2: AGG CUU UUA AAG CCA UAU AC 3′ (deletion size: ~0.200 kb); *MLH1*: gRNA1: 5′ ACUGAUAGAAAUUGGAUGUG 3′, gRNA2: 5′ CUU CAC UGA GUA GUU UGC AU 3′ (deletion size ~0.06 kb). The chemical modifications: 2′‐O‐Methyl at the three first and last bases, and 3′ phosphorothioate bonds between the first three and last two bases, were introduced into the gRNAs in order to provide superior editing in the organoid lines used (Synthego). No suitable gRNAs were available for PMS2. The Cas9 2NLS Nuclease was purchased from Synthego. Organoid lines were electroporated by gRNA and Cas9 (1:3 ratio) using the Neon Transfection System (Thermo Fisher, MPK5000S). Electroporated cells were allowed to grow for approximately 7 days prior to DNA and RNA harvesting. CRISPR editing of each MMR gene was performed in paired organoid lines derived from right and left colon biopsies of three different individuals. Genomic DNA was purified using the QIAamp DNA Mini Kit (Qiagen) and DNA deletions were confirmed with PCR amplifications *MSH2*: forward primer: 5′ CAG CTT CCA TTG GTG TTG TG 3′, reverse primer: 5′ GGG GAG AAA AGA TCT GAG GT 3′ (amplicon size ~0.45 kb); *MSH6*: forward: 5′ ATC TGA GGG GGA TTG GTT GC 3′, reverse: 5′ CAT GCC AGG CTG TTG ATG TC 3′ (amplicon size ~2.0 kb); *MLH1*: forward 5′ GAG GAC CTC AAA TGG ACC AA 3′, reverse 5′ AAC CAA ACT TTG CCA TGA GG 3′ (amplicon size ~0.39 kb). CRISPR‐Cas9 editing of *MSH4* was also performed. However, we were unable to achieve successful editing of these genes using the gRNAs tested (data not shown). The evaluation of *MSH5* expression (partner to *MSH4*) was restricted to right and left colon organoids of one individual following *MSH2* CRISPR editing and was considered as a negative control. This individual was chosen based on the availability of sufficient RNA in both left and right colon organoids for control and CRISPR‐edited lines. Experiments were performed in triplicate to address reproducibility.

### 2.18 Quantitative Real‐Time PCR

RNA was isolated using Trizol reagent (Thermo Fisher: 15596026) and cDNA was synthesized from 2 μg of total RNA using the High‐Capacity Reverse Transcriptase cDNA kit (Thermo Fisher: 4368813). Quantitative real‐time polymerase chain reaction (qRT‐PCR) was performed using the Superscript III kit for RT‐PCR (Thermo Fisher: 18080051) and amplified using TaqMan assays for the following genes: *MSH2* (Hs00953527_m1), *MSH4* (Hs00172489_m1), *MSH5* (Hs00159268), *MSH6* (Hs00943000_m1), and *MLH1* (Hs00179866_m1), and the internal control, Glucuronidase Beta (*GUSB*; Hs00939627_m1). Statistical significance was determined by controlling for subject identifiers in a linear mixed effects regression model using the *lmerTest* package.

## 3 RESULTS

### 3.1 Outline of study to define cancer risk genes

**FIGURE 1:**
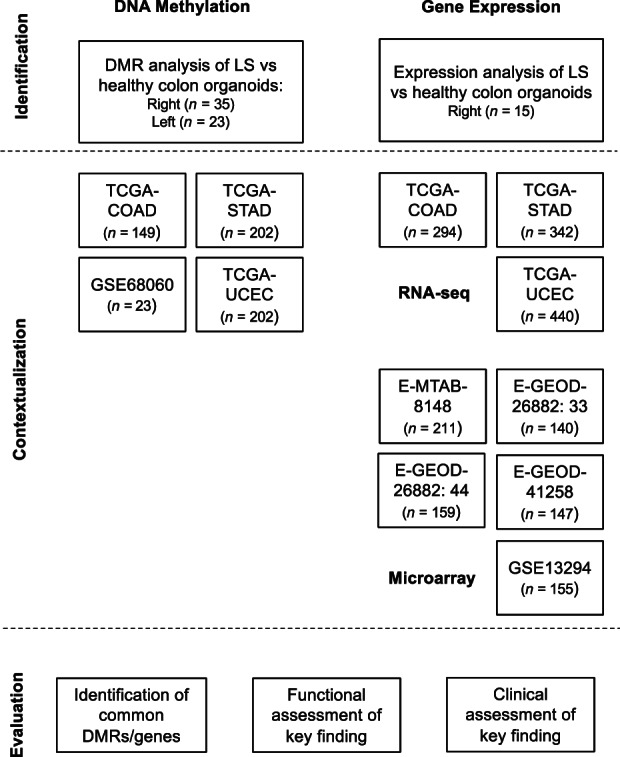
Diagram to show the role of the various cohorts used within each of the various stages of the current study. Broadly, the study can be split into data generated from two omic layers (gene expression and DNA methylation) and three stages (identification, contextualization, and evaluation).

Although much attention has been paid to the evaluation of omic markers of disease, a better understanding of genes occurring prior to disease onset may help in the identification of at‐risk populations through simple, targeted screening approaches. An overview of our approach to define risk genes associated with MSI‐H tumors and dMMR is outlined in Figure 1. Various drug combinations have been shown to be effective for MSS and MSI‐H tumor biology^50^, ^51^, ^52^, ^53^, ^54^, ^55^, ^56^, ^57^, ^58^, ^59^, ^60^, ^61^, ^62^, ^63^, ^64^, ^65^, ^66^, ^67^, ^68^, ^69^, ^70^, ^71^, ^72^ (Table S1). Drug use was not considered a covariate in our regression analysis.

### 3.2 Comparing DNA methylomes of organoids from normal colons of Lynch syndrome and healthy subjects

**FIGURE 2:**
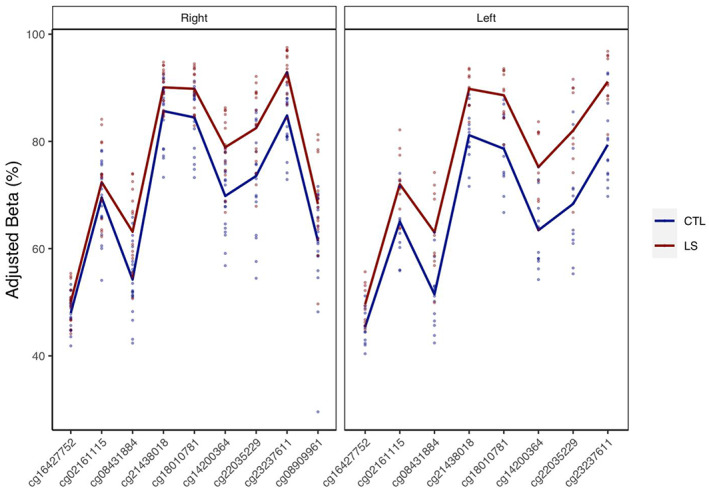
DMR plot of *MSH4* region found to be significantly different between LS and healthy subjects in right colon organoids and left colon organoids. Beta values were plotted following adjustment for technical variation by COMBAT.

Aberrant levels of DNA methylation are a hallmark of CRC.^73^ To determine whether organoids derived from the normal colons of LS versus healthy subjects displayed significant epigenetic profiles relevant to disease, we first performed an epigenome‐wide analysis of DNA methylation (Illumina Infinium MethylationEPIC array, EPIC) in 58 organoid lines derived from 42 unique individuals (Table S2). Individual DMR analyses were carried out in right and left colon organoid subsets to identify differences between LS and healthy subjects. In our analysis of right colon organoids (*n* = 35), we identified 241 DMRs that survived false discovery rate (FDR) correction (Table S3). This included a number of genes not previously associated with LS, such as DNA hypermethylation of *MSH4* (FDR = 2.31E^−06^) (Figure 2). Notably, *MSH4* is a member of the MutS family of MMR genes. However, it has not previously been implicated in LS and has been reported to play a role exclusively in meiosis.^74^ A similar analysis for LS versus healthy colon organoids was performed in a largely overlapping cohort of left colon organoids (*n* = 23). Here, we identified 202 DMRs (Table S4). Of these, only 25 were also significant in right colon organoids (Table 1), including LS‐specific DNA hypermethylation of the *MSH4* locus (FDR = 6.72E^−04^).

**TABLE 1:** (A) Summary of DMRs identified in right colon organoids of LS versus healthy subjects that were also significant in left colon organoids. (B) Summary of DMRs in identified in analysis of left colon organoids of LS versus healthy subjects that were also significant in right colon organoids.

Chr	Start (bp)	End (bp)	No. of CpGs	Stouffer *p* value	FDR	Mean difference (Beta %)	Overlapping genes
(A)							
8	86,350,278	86,351,195	12	1.86E‐15	2.26E‐13	−0.062	*CA3*
8	101,117,902	101,118,899	14	5.30E‐13	2.16E‐11	−0.096	*RGS22*
11	86,085,026	86,086,101	11	6.25E‐12	1.91E‐10	−0.103	*CCDC81*
13	20,805,094	20,805,895	11	4.59E‐10	4.87E‐09	−0.093	*GJB6*
10	103,603,292	103,604,528	13	8.55E‐09	6.73E‐08	−0.051	*KCNIP2*
2	177,042,493	177,043,501	8	8.97E‐09	6.84E‐08	0.117	*AC009336.24, HOXD‐AS1*
14	95,047,591	95,048,198	11	7.09E‐08	3.39E‐07	−0.070	*SERPINA5*
1	76,262,302	76,262,984	9	6.35E‐07	2.31E‐06	0.076	*MSH4*
**6**	**28,956,226**	**28,956,804**	**19**	**4.98E‐06**	**1.25E‐05**	**−0.104**	***HCG16***
7	96,636,746	96,638,339	7	6.22E‐06	1.51E‐05	−0.083	*DLX6, DLX6‐AS1, DLX6‐AS2*
6	31,894,990	31,895,598	8	1.31E‐05	2.73E‐05	−0.066	*C2, CFB*
16	10,970,709	10,971,250	9	1.44E‐05	2.94E‐05	−0.076	*CIITA, RP11‐876 N24.2*
**10**	**7,450,813**	**7,452,242**	**11**	**2.48E‐05**	**4.61E‐05**	**0.071**	***SFMBT2***
12	113,415,883	113,416,518	8	2.51E‐05	4.61E‐05	−0.072	*OAS2, RP1‐71H24.1*
2	84,743,142	84,743,935	12	5.84E‐05	9.69E‐05	−0.096	*DNAH6*
8	70,378,354	70,378,760	7	9.38E‐05	1.46E‐04	−0.091	NA
14	104,689,833	104,690,244	7	2.21E‐04	3.17E‐04	−0.115	*RP11‐260 M19.2*
10	118,429,452	118,429,952	7	5.38E‐04	7.19E‐04	−0.100	*RP11‐498B4.5, C10orf82*
2	30,669,385	30,670,170	11	1.63E‐03	1.96E‐03	0.054	*LCLAT1*
5	140,792,511	140,792,918	9	2.04E‐03	2.42E‐03	−0.100	*PCDHGA1, PCDHGB1*
15	39,871,808	39,872,401	8	2.30E‐03	2.70E‐03	−0.103	NA
7	101,005,832	101,006,308	11	3.59E‐03	4.07E‐03	−0.068	*COL26A1*
6	29,943,209	29,943,480	8	4.50E‐03	5.01E‐03	−0.087	*HCG9*
11	41,481,292	41,481,891	8	4.54E‐03	5.04E‐03	−0.083	*LRRC4C*
3	46,759,335	46,759,977	9	0.022	0.023	−0.050	*PRSS50*
(B)							
8	86,350,278	86,351,195	12	3.14E‐13	2.25E‐11	−0.086	*CA3*
2	84,742,902	84,743,935	13	7.12E‐09	1.09E‐07	−0.090	*DNAH6*
2	177,042,493	177,043,501	8	1.01E‐08	1.21E‐07	0.163	*AC009336.24, HOXD‐AS1*
**10**	**7,450,813**	**7,452,242**	**11**	**7.11E‐08**	**5.66E‐07**	**−0.075**	***SFMBT2***
8	101,117,902	101,118,899	14	1.35E‐07	9.64E‐07	−0.086	*RGS22*
15	39,871,808	39,872,463	9	7.06E‐07	3.89E‐06	−0.165	NA
3	46,758,813	46,759,977	11	6.14E‐06	2.36E‐05	−0.082	*PRSS50*
14	104,688,607	104,690,244	10	1.04E‐05	3.34E‐05	−0.110	*RP11‐260 M19.2*
16	10,970,709	10,971,250	9	1.67E‐05	4.93E‐05	−0.094	*CIITA, RP11‐876 N24.2*
7	96,635,336	96,637,365	14	1.93E‐05	5.54E‐05	−0.058	*DLX6, DLX6‐AS1, DLX6‐AS2*
2	30,669,597	30,670,304	11	3.92E‐05	9.80E‐05	0.052	*LCLAT1*
10	118,429,452	118,429,952	7	8.56E‐05	1.88E‐04	−0.098	*RP11‐498B4.5, C10orf82*
11	86,085,366	86,086,101	10	8.71E‐05	1.89E‐04	−0.086	*CCDC81*
12	113,415,883	113,416,518	8	1.25E‐04	2.56E‐04	−0.064	*OAS2, RP1‐71H24.1*
1	76,262,302	76,262,857	8	3.78E‐04	6.72E‐04	0.074	*MSH4*
6	31,895,179	31,895,598	7	4.57E‐04	7.93E‐04	−0.097	*C2, CFB, CFB*
13	20,805,196	20,806,746	18	6.21E‐04	1.05E‐03	−0.060	*GJB6*
5	140,792,511	140,792,918	9	7.29E‐04	1.20E‐03	−0.100	*PCDHGA1‐10, PCDHGB1‐6,*
**6**	**28,956,226**	**28,956,804**	**19**	**8.33E‐04**	**1.34E‐03**	**0.078**	***HCG16***
6	29,943,209	29,943,677	9	1.08E‐03	1.68E‐03	−0.152	*HCG9*
7	101,005,832	101,007,603	16	1.24E‐03	1.88E‐03	−0.056	*COL26A1*
8	70,378,354	70,378,760	7	1.30E‐03	1.94E‐03	−0.093	NA
10	103,603,292	103,604,222	12	3.91E‐03	5.26E‐03	−0.051	*KCNIP2*
11	41,481,148	41,481,655	9	6.31E‐03	8.12E‐03	−0.076	*LRRC4C*
14	95,047,591	95,047,880	8	0.037	0.039	−0.065	*SERPINA5*

*Note*: Bold font indicates genes that were discordant for the direction of effect. Positive mean difference values indicate an increased average beta value for that region in LS versus healthy controls. Genes are ordered by decreasing significance.

### 3.3 Bisulfite sequencing of *MSH4* locus

We bisulfite‐sequenced a 587 bp region (chr1: 76262302–76262889) and generated data for 27 individual cytosines across 15 samples (Table 2) to technically validate the *MSH4* locus in a subset of right colon organoids. Eighteen cytosines were significantly different between LS and healthy controls (n_LS_ = 7; n_CTL_ = 8). All but one of the 27 sites were hypermethylated in the LS colon. Of the eight sites that overlapped with cytosines on the Illumina EPIC array, six were significant and followed the same direction of effect.

**TABLE 2:** Summary of Bisulfite‐Seq results for *MSH4* DMR on chromosome 1.

Position (bp)	Present on EPIC array	P	Q	Mean difference (% Beta)	M.SssI average (% beta)
76,262,648	X	6.21E‐04	0.004	29.360	0.959
76,262,505	X	0.024	0.014	22.649	0.931
76,262,537	X	0.021	0.014	22.249	0.931
76,262,543	X	0.015	0.014	25.856	0.934
76,262,560		0.029	0.014	22.093	0.884
76,262,563		0.019	0.014	19.604	0.951
76,262,566		0.021	0.014	24.327	0.920
76,262,568		0.020	0.014	25.378	0.946
76,262,583		0.025	0.014	24.901	0.920
76,262,591		0.035	0.014	22.500	0.949
76,262,594	X	0.035	0.014	21.870	0.964
76,262,626		0.026	0.014	24.337	0.955
76,262,740		0.035	0.014	18.566	0.961
76,262,758		8.17E‐03	0.014	22.074	0.965
76,262,769		5.82E‐03	0.014	22.694	0.967
76,262,801		0.031	0.014	20.592	0.968
76,262,794		0.041	0.015	17.708	NA
76,262,579		0.059	0.018	21.028	0.924
76,262,699		0.053	0.018	20.093	0.961
76,262,302	X	8.97E‐03	0.043	12.537	0.928
76,262,726		0.266	0.078	10.386	0.912
76,262,729		0.296	0.083	11.685	0.959
76,262,714		0.399	0.107	10.218	0.968
76,262,711		0.425	0.109	8.783	0.969
76,262,723		0.559	0.138	7.659	0.964
76,262,889		0.145	0.195	−7.218	0.693
76,262,373	X	0.501	NA	1.716	0.775

*Note*: Positive beta differences indicated DNA hypermethylation of the cytosine at that site in LS versus healthy colon organoids. Probes were cross‐referenced to their presence on the EPIC array (X) following initial filtering in the right colon organoid analysis.

### 3.4 Analysis of DMRs in MSI‐H tumor data sets

To define the relationship between LS DMRs and DNA methylation associated with MSI‐H tumors, we first aimed to define consistent differences occurring between MSI‐H and MSS/MSI‐L tumors across three gastrointestinal cancer cohorts (see Methods). We identified 2519 DMRs in TCGA‐COAD (*n* = 149), 3986 DMRs in GSE68060^75^ (*n* = 23), and 3144 in TCGA‐STAD (n = 202) data sets (Table S5). To determine the confidence of the association between these DMRs and tumor biology, DMRs were grouped into five categories (see Methods). Next, we related these tumor DMRs to those previously identified in our analysis of LS versus healthy colon organoids. Of the 241 DMRs identified in right colon organoids, 27 were found to be associated with MSI‐H status, with *MSH4* being the highest confidence DMR identified (Table 3). Of the 202 DMRs identified in our analysis of left colon organoids, 45 were found to be associated with MSI‐H status (Table 3), *MSH4*, as well as three additional highest confidence DMRs: chr14:24,779,793‐24,780,926 which corresponded to multiple genes; chr17:37,123,638–37,124,209 (F‐box protein 47 (*FBXO47*)) and chr15:51,973,083–51,973,591 (Secretogranin III (*SCG3*)).

**TABLE 3:** (A) Summary of DMRs in right sided colon organoid of LS subjects with some level of replication in gastrointestinal cancer MSI‐H datasets. (B) Summary of DMRs in left sided colon organoid of LS subjects with some level of replication in gastrointestinal cancer MSI‐H datasets.

Chr	Start (bp)	End (bp)	No. of CpGs	Stouffer *p* value	FDR	Mean difference (% Beta)	Overlapping genes	Confidence
(A)								
14	38,090,803	38,092,175	11	2.76E‐11	5.60E‐10	−0.053	*TTC6*	Low
7	150,652,702	150,653,449	10	9.08E‐11	1.23E‐09	−0.066	*KCNH2*	Medium‐Colon
6	28,557,047	28,558,113	23	2.24E‐08	1.40E‐07	−0.069	*RP5‐1186 N24.3, SCAND3*	Medium‐Colon
14	99,786,193	99,787,620	8	2.84E‐08	1.73E‐07	−0.102	NA	Low
**1**	**87,616,687**	**87,617,811**	**10**	**3.67E‐08**	**1.99E‐07**	**0.114**	**RP5‐1052I5.2, LINC01140, AL139139.1**	**Medium**
13	50,701,050	50,703,841	17	4.51E‐08	2.29E‐07	−0.065	*DLEU1*	Low
4	111,549,880	111,552,353	14	2.59E‐07	1.07E‐06	−0.12	*PITX2*	Medium‐Colon
**1**	**76,262,302**	**76,262,984**	**9**	**6.35E‐07**	**2.31E‐06**	**0.076**	***MSH4***	**Highest**
13	50,706,583	50,708,043	11	8.04E‐07	2.69E‐06	−0.081	*DLEU1*	Low
**5**	**83,016,630**	**83,017,184**	**7**	**2.28E‐06**	**6.38E‐06**	**0.108**	***HAPLN1***	**High**
1	91,300,215	91,300,559	7	3.05E‐06	8.10E‐06	−0.162	*RP4‐665 J23.1*	Medium‐Colon
**6**	**85,473,238**	**85,474,803**	**15**	**4.82E‐06**	**1.23E‐05**	**0.067**	***TBX18***	**Low**
**5**	**140,430,412**	**140,431,329**	**10**	**5.06E‐06**	**1.26E‐05**	**0.106**	***PCDHB1***	**Low**
19	51,017,855	51,018,414	7	8.72E‐06	1.99E‐05	0.058	*ASPDH*	High
6	28,226,567	28,227,482	15	1.54E‐05	3.14E‐05	−0.06	*NKAPL, ZKSCAN4*	Low
**20**	**62,687,071**	**62,688,717**	**13**	**2.31E‐05**	**4.48E‐05**	**0.054**	***TCEA2, RP13‐152O15.5***	**Low**
12	113,415,883	113,416,518	8	2.51E‐05	4.61E‐05	−0.072	*OAS2, RP1‐71H24.1*	Low
**22**	**31,535,981**	**31,536,996**	**7**	**3.88E‐05**	**6.82E‐05**	**−0.074**	***PLA2G3***	**Medium**
14	54,686,184	54,686,833	7	6.71E‐05	1.10E‐04	−0.075	NA	Low
12	115,104,274	115,105,519	10	9.38E‐05	1.46E‐04	−0.066	NA	Low
1	36,042,433	36,043,401	7	1.10E‐04	1.67E‐04	−0.082	*TFAP2E, RP4‐728D4.2*	Low
5	151,066,484	151,067,485	7	2.44E‐04	3.44E‐04	−0.066	*CTB‐113P19.1, SPARC*	Low
**17**	**46,810,447**	**46,810,934**	**7**	**1.58E‐03**	**1.91E‐03**	**0.058**	***CTD‐2377D24.4***	**Medium**
8	144,790,089	144,790,410	8	3.15E‐03	3.61E‐03	−0.075	*ZNF707, CCDC166*	Low
6	29,943,209	29,943,480	8	4.50E‐03	5.01E‐03	−0.087	*HCG9*	Medium‐Colon
12	81,471,194	81,472,177	15	5.17E‐03	5.70E‐03	−0.085	*ACSS3*	Medium‐Colon
7	5,111,488	5,112,654	14	6.83E‐03	7.41E‐03	−0.067	*RBAK‐RBAKDN, RBAKDN*	Low
(B)								
7	130,130,122	130,132,453	48	6.35E‐29	1.37E‐26	−0.088	*MEST*	Low
3	42,976,956	42,978,318	14	2.74E‐10	7.35E‐09	0.075	*KRBOX1, KRBOX1, KRBOX1‐AS1*	Medium
16	66,612,774	66,613,407	12	7.89E‐09	1.13E‐07	−0.081	*CKLF‐CMTM1, CMTM1, CMTM2*	Low
7	4,764,228	4,765,362	7	8.52E‐09	1.14E‐07	−0.133	*FOXK1*	High
10	81,001,957	81,003,657	7	9.52E‐09	1.20E‐07	−0.218	*ZMIZ1*	Low
17	48,545,805	48,546,620	12	2.93E‐08	2.87E‐07	−0.110	*ACSF2, CHAD*	High
11	63,766,546	63,768,794	10	3.16E‐08	2.96E‐07	−0.113	*OTUB1, MACROD1*	Low
14	24,779,793	24,780,926	15	6.44E‐08	5.54E‐07	−0.090	*LTB4R2, LTB4R2, LTB4R, CIDEB*	Highest
2	241,497,060	241,497,975	10	9.90E‐08	7.34E‐07	−0.087	*ANKMY1*	Low
21	45,148,332	45,149,373	7	4.25E‐07	2.69E‐06	−0.129	*PDXK*	Low
17	76,354,621	76,355,674	7	5.93E‐07	3.36E‐06	−0.092	*SOCS3*	Low
2	54,086,854	54,087,552	13	7.49E‐07	4.03E‐06	−0.088	*GPR75‐ASB3, ASB3, GPR75*	Low
19	58,545,001	58,545,837	11	1.54E‐06	7.20E‐06	−0.202	*ZSCAN1*	Medium‐Colon
2	236,442,817	236,444,284	12	1.58E‐06	7.22E‐06	−0.129	*AGAP1*	Medium
13	28,496,413	28,498,956	13	1.64E‐06	7.33E‐06	−0.051	*PDX1*	High
2	239,140,032	239,140,369	7	6.50E‐06	2.45E‐05	−0.141	*AC096574.4, AC016757.3*	Low
**19**	**40,724,308**	**40,725,336**	**8**	**1.19E‐05**	**3.65E‐05**	**0.055**	**NA**	**Low**
**12**	**132,899,065**	**132,900,761**	**9**	**1.74E‐05**	**5.06E‐05**	**0.052**	***GALNT9***	**Medium**
12	52,626,062	52,627,438	10	2.19E‐05	6.13E‐05	−0.070	*KRT7*	High
2	113,992,694	113,993,313	8	2.22E‐05	6.13E‐05	−0.119	*PAX8‐AS1, PAX8*	Low
2	119,599,067	119,600,002	8	2.46E‐05	6.68E‐05	−0.093	*EN1*	Medium‐Colon
4	1,202,272	1,203,168	18	3.77E‐05	9.65E‐05	−0.157	*SPON2*	Low
21	38,077,042	38,077,971	8	4.02E‐05	9.93E‐05	−0.088	*SIM2*	High
16	67,199,335	67,200,181	7	4.08E‐05	9.98E‐05	−0.068	*RP11‐5A19.5, HSF4*	High
5	140,578,896	140,579,644	10	6.82E‐05	1.61E‐04	−0.093	*PCDHB11*	Low
7	93,520,024	93,520,566	12	7.23E‐05	1.64E‐04	−0.085	*GNGT1, AC002076.10, TFPI2*	Medium‐Colon
19	291,986	292,498	13	8.36E‐05	1.85E‐04	0.057	NA	Low
17	73,583,839	73,584,617	9	1.07E‐04	2.31E‐04	−0.084	*MYO15B*	Medium‐Colon
11	57,193,703	57,194,498	8	1.17E‐04	2.49E‐04	−0.059	*SLC43A3*	Low
12	113,415,883	113,416,518	8	1.25E‐04	2.56E‐04	−0.064	*OAS2, RP1‐71H24.1*	Low
17	37,123,638	37,124,209	10	1.40E‐04	2.74E‐04	0.057	*FBXO47*	Highest
7	42,267,200	42,267,747	8	1.97E‐04	3.66E‐04	−0.117	*GLI3*	Medium‐Colon
**1**	**76,262,302**	**76,262,857**	**8**	**3.78E‐04**	**6.72E‐04**	**0.074**	***MSH4***	**Highest**
6	28,956,226	28,956,804	19	8.33E‐04	1.34E‐03	0.078	*HCG16*	Low
6	29,943,209	29,943,677	9	1.08E‐03	1.68E‐03	−0.152	*HCG9*	Medium‐Colon
**6**	**30,070,886**	**30,071,612**	**26**	**1.44E‐03**	**2.11E‐03**	**0.059**	***TRIM31***	**Low**
1	67,600,428	67,601,159	8	1.85E‐03	2.65E‐03	−0.072	*C1orf141*	Low
**15**	**51,973,083**	**51,973,591**	**7**	**5.83E‐03**	**7.55E‐03**	**0.068**	***SCG3***	**Highest**
**7**	**96,651,915**	**96,652,481**	**8**	**8.59E‐03**	**0.011**	**−0.072**	***DLX5***	**Medium‐Colon**
17	40,439,433	40,439,892	8	0.016	0.019	−0.071	*STAT5A*	Low
**3**	**116,164,242**	**116,164,943**	**8**	**0.017**	**0.020**	**0.057**	***LSAMP***	**Medium**
11	65,816,521	65,816,985	7	0.022	0.025	−0.058	*GAL3ST3*	Low
13	37,005,273	37,005,582	10	0.025	0.028	−0.117	NA	Medium‐Colon
17	9,728,972	9,729,424	9	0.026	0.029	0.053	*GLP2R*	Low
**2**	**202,483,799**	**202,484,156**	**7**	**0.042**	**0.044**	**0.068**	***ALS2CR11***	**Low**

*Note*: Bold font indicates DMRs that were significant and concordant with overlapping DMRs in our analysis of MSI‐H versus MSS in the TCGA‐UCEC DNA methylation data set. Positive values represent DNA hypermethylation of that DMR within LS (vs normal) or MSI‐H (vs MSS) samples.

A subset of endometrial cancers also exhibits MSI‐H, so we downloaded and processed DNA methylation data to determine if similarities exist with gastrointestinal tumors: TCGA‐UCEC (see Methods). Eight right‐side (Table 3, bold font) and eight left‐side (Table 3, bold font) DMRs identified in our analysis of LS versus healthy colon organoids overlapped MSI‐H‐related DMRs in TCGA‐UCEC, including MSH4. Together, these data reveal a consistent role for differential methylation of *MSH4* in MSI‐H tumors across tumor locations.

### 3.5 Evaluation of expression of *MSH4* and other DMR candidate genes in LS and tumor data sets

The governance of gene expression is a primary role of DNA methylation. To determine the relationship between gene expression and DNA methylation of *MSH4‐* and other MSI‐H‐related DMRs, we performed the analysis of three publicly available TCGA cohorts with available RNA‐seq and MSI‐H data (TCGA‐COAD, TCGA‐STAD, and TCGA‐UCEC) using *DESeq2*,^40^ while adjusting for cell composition (see Methods). Our analysis in TCGA‐COAD has previously been reported.^36^ As our primary goal was to better define the role of LS DMRs in cancer risk, we limited our analysis to genes corresponding to DMRs identified in Table 3 (Table S6). Of the highest confidence DEGs identified in right or left colon organoids, a reduction in *SCG3* expression in MSI‐H tumors was observed for TCGA‐STAD (FDR = 7.44E‐03) and TCGA‐COAD (P = 0.042) tumors. A reduction in *FBXO47* expression in MSI‐H tumors was also observed in TCGA‐STAD (FDR = 1.25E‐03) and TCGA‐UCEC tumors (FDR = 2.55E‐03). *MSH4* was increased in MSI‐H versus MSS/MSI‐L tumors in all three data sets: TCGA‐COAD (FDR = 1.46E^−13^), TCGA‐STAD (FDR = 7.32E^−17^), and TCGA‐UCEC (FDR = 4.60E^−07^). We confirmed this *MSH4* finding in a meta‐analysis of MSI‐H vs MSS tumors using five large (*n* > 100) microarray colon data sets (Table S7) as well as in an individual analysis of each cohort: GEO26682_33 (P = 1.65E^−07^), GEO26682_44 (P = 5.26E^−13^), GEO41258 (P = 9.87E^−04^), E‐MTAB‐8148 (P = 3.09E^−12^), and GEO13294 (P = 2.84E^−14^).^42^, ^44^, ^45^, ^46^, ^47^ Neither *SCG3* nor *FBXO47* was identified in our meta‐analysis, though a nominal reduction for *SCG3* was observed in E‐MTAB‐8148 (P = 0.031).

We also performed an RNA‐seq analysis of a largely overlapping cohort of right‐sided colon organoids of LS (*n* = 7) versus healthy subjects (*n* = 9). We identified 811 nominal DEGs (*p* < 0.05; Table S7). Limited overlap was found between DMRs associated with the MSI‐H phenotype. However, our analysis did reveal an increase in *MSH4* (*p* = 7.04E^−03^) expression in LS versus healthy subjects. *RBAK downstream neighbor* (*RBAKDN*, *p* = 0.027) expression was also increased in LS colon organoids. *RBAKDN* is a low confidence DMR that was also found to be hypomethylated (Tabe S5) and concomitant with increased gene expression (Table S6) specifically in TCGA‐COAD MSI‐H tumors.

### 3.6 CRISPR deletion of MMR genes leads to increased expression of *MSH4*

**FIGURE 3:**
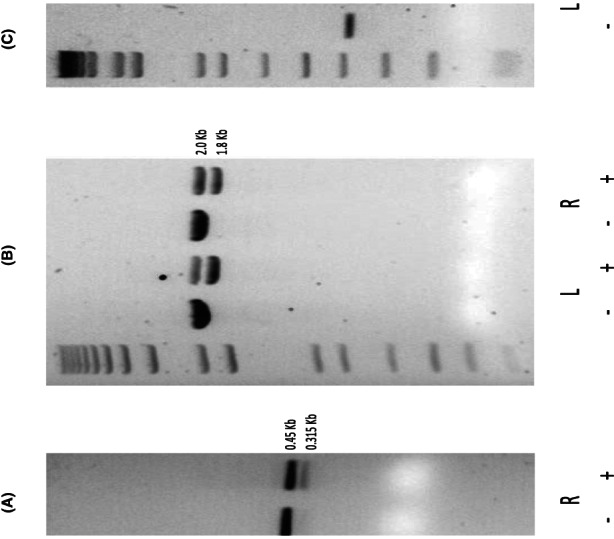
CRISPR‐Cas9 targeting of *MSH2*, *MSH6,* and *MLH1*. DNA gel electrophoresis showing genome editing of (A) *MSH2*, (B) *MSH6*, and (C) *MLH1*. For *MSH2*, the 0.315 kb band demonstrates targeted deletion of the gene, whereas the 0.45 kb band represents part of the *MSH2* gene amplified by PCR. For *MSH6*, a 1.8 kb band demonstrates a targeted deletion, whereas a 2.0 kb band represents part of *MSH6* gene amplified by PCR. For *MLH1*, a 0.33 kb band demonstrates targeted deletion of the gene, and a 0.39 kb band represents part of *MLH1* amplified by PCR. Left (L) and right (R) colon organoids were tested. (−): cells electroporated with mock conditions; (+): cells electroporated with cas9 vector and guide RNA vectors targeting the gene of interest. A 1 kb ladder was used to measure fragment length.

To validate *MSH4* as a marker of dMMR we performed CRISPR‐mediated deletion of LS‐related MMR genes (*MSH2*, *MLH1*, and *MSH6*) in matched right and left colon organoids from three healthy subjects (see Methods). *PMS2* was not considered because of a highly homologous pseudogene. We used gRNAs to delete approximately 135 bp, 200 bp, and 60 bp fragments from *MSH2*, *MSH6*, and *MLH1*, respectively (Figure 3A–C; Figure S1A‐C). Deletion efficiency in the organoid lines was assessed by PCR using primers designed to amplify approximately 450 bp, 2 kb, and 390 kb genomic regions for *MSH2*, *MSH6*, and *MLH1*, respectively. Mock‐treated cells yielded a single 450 bp band (*MSH2*), a 2 kb band (*MSH6*), and a 390 bp band (*MLH1*), corresponding to an unedited genomic fragment, whereas CRISPR‐Cas9‐transfected cells yielded two bands: the unedited fragments and the edited 315 bp (MSH2), 1.8 kb (*MSH6*), and 330 bp (*MLH1*) fragments. DNAs were harvested and used to assess the deletion efficiency. RNAs were isolated from the same cellular pools and the expression of each target gene was tested by qPCR. A significant increase in *MSH4* expression was observed in right colon organoids following deletion of *MLH1* (P = 6.17E^−03^) or *MSH2* (P = 8.92E^−03^). A nonsignificant increase in *MSH4* expression was also observed in two of three right colon organoid lines following *MSH6* knockdown. MSH5 forms a heterodimer with MSH4, but increased *MSH4* expression was seen even in the absence of any significant change in *MSH5* expression (Figure S2), in *MSH2* knockdowns. However, a medium correlation was observed between *MSH4* and *MSH5* expression across both left and right colon organoids (*r* = 0.34). A nonsignificant increase in *MSH4* expression was observed in matched, left‐sided colon organoids following *MLH1* (*p* = 0.57), *MSH2* (*p* = 0.097), or *MSH6* (*p* = 0.64) CRISPR deletion. *MSH4* was increased in all three lines following the *MSH2* knockdown, but this did not reach statistical significance.

### 3.7 Defining a clinical role for *MSH4* expression

**FIGURE 4:**
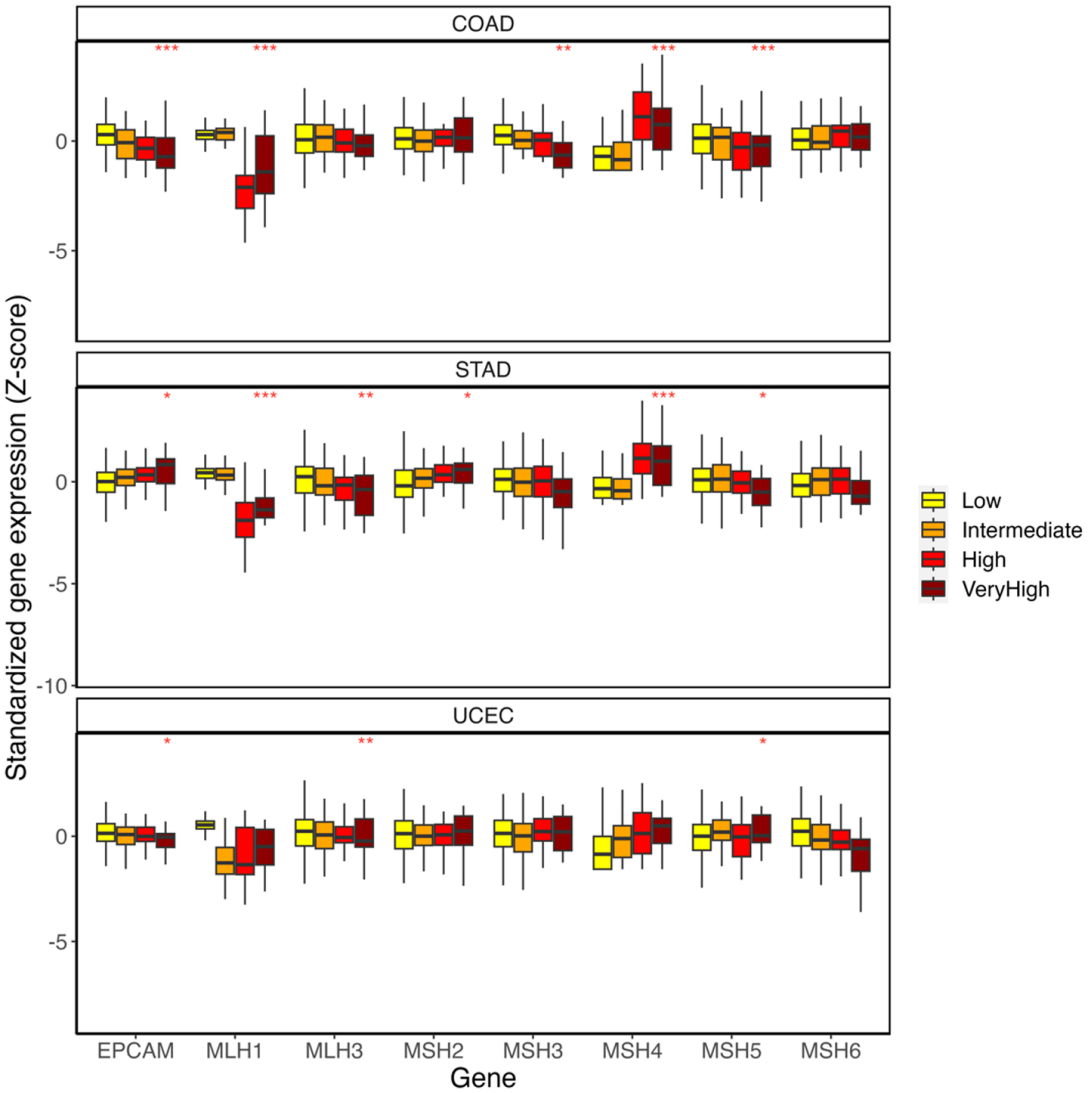
Comparison of relative expression scores of genes related to MMR and TMB. TMB was grouped into four categories: low (≤5), intermediate (>5, ≤20), high (>20, ≤50), and very high (>50). Significance denoted by *(*p* < 0.05), **(*p* < 0.005), and ***(*p* < 0.0005).

We have established *MSH4* as a consistent feature of dMMR. To study its potential clinical role, Z‐score gene expression values were downloaded from cBioPortal.^48^ Individuals with MSI‐H have been shown to present with a TMB. We, therefore, also downloaded individual‐level data on this measure from cBioPortal.^48^ Linear regressions of MMR genes on TMB revealed that *MSH4* expression was most significantly associated with TMB compared with all MMR genes (Figure 4) in TCGA‐COAD (P = 9.15E‐11), and secondmost (after *MLH1*) in TCGA‐STAD (P = 5.72E^−09^). An association between *MSH4* and nonsynonymous TMB was only identified in TCGA‐UCEC following the removal of individuals defined by POLE subtype (P = 8.63E^−03^).

Finally, Cox proportional hazards models were fitted to interrogate the relationship between survival outcomes and *MSH4* expression (low, high as defined by median cutoff) while accounting for adjustment covariates. Here, we found that high *MSH4* expression trended toward poorer OS in TCGA‐COAD (*p* = 0.053), TCGA‐STAD (*p* = 0.068), and TCGA‐UCEC (*p* = 0.064).

## 4 DISCUSSION

CRC is a highly heterogeneous disease and fundamental differences in CRC tumor biology exist between MSI‐H and MSS CRC subgroups.^36^ For example, MSI‐H tumors are more mucin‐rich and reveal a higher number of tumor‐infiltrating lymphocytes than MSS tumors.^4^ MSI‐H tumors also display a higher tumor mutational burden (TMB) than MSS.^76^ Within MSI‐H tumors, nonhereditary MSI‐H tumors are predominately right‐sided, whereas LS‐related MSI‐H tumors occur at similar rates in both the right and left colon.^77^ Despite these differences, we have shown that several epigenetic and transcriptomic similarities are present between nonhereditary MSI‐H tumors and epithelial cells derived from the normal colon of LS individuals. Understanding the molecular mechanisms contributing to MSI‐H tumors may help improve personalized treatment options. Identifying disease‐relevant genes that precede CRC onset in individuals at high risk of developing cancer such as LS may also lead to improved diagnoses, for example, in the absence of a known germline mutation, and improved outcomes. Therefore, we propose that the MSI‐H‐related differences in normal colon organoids of cancer‐free LS subjects identified here have the potential to serve as useful clinical biomarkers for the at‐risk LS population. Most notably, our findings support an important correlation between *MSH4* DNA methylation and gene expression occurring in dMMR/MSI‐H across different cancer types and in both hereditary/nonhereditary conditions. Our findings strongly support the role of *MSH4* in dMMR. Given the important role of dMMR in numerous cancers, future studies should aim to determine whether differences in *MSH4* expression may be used in combination with other screening‐based approaches to identify and provide supporting individuals in the general population with dMMR. Such a strategy may be particularly useful for individuals suspected of LS, with no known causal variant.

This strategy may be particularly important given that our findings implicate some relationship between increased *MSH4* expression and OS in each TCGA cohort. Though no finding reached statistical significance (*p* < 0.05), each trended in a direction to show that individuals with higher than the median expression of *MSH4* expression displayed poorer OS after adjustment for factors such as tumor stage and MSI status. However, given the high degree of missingness of survival data and the relatively limited number of MSI‐H tumors in each cancer type, this analysis was limited to tumors across both MSI‐H and MSS subgroups. It remains possible that the effects on survival may be greater in one subgroup and larger studies should consider this when conducting future analysis.

The sample size of our primary analysis was relatively small. Further, cancer biology is highly heterogeneous. As such, we focused on differences that were consistent across data sets and omic layers. The most consistent association we found in LS and between MSI‐H and MSS tumor status was *MSH4*. *MSH4* was the only DMR identified in both right and left colon organoids of LS subjects that were of “highest confidence” in our subsequent analysis of MSI‐H vs MSS/MSI‐L tumors. This finding was validated using previously published data in MSI‐H tumors and across omic layers. *MSH4* has not previously been implicated in LS and previous studies have considered it only to play a role in meiosis.^74^ Interestingly, a recent study in bladder cancer implies some genetic evidence for an association between MSH4 and MSI‐H.^78^ To the best of our knowledge, a role for *MSH4* in LS has yet to be defined.

To confirm the correlation between *MSH4* expression and dMMR, we performed CRISPR‐Cas9 deletion of three known LS‐related MMR genes and measured the effect on *MSH4* gene expression. To reduce the potential of other MMR genes driving any observed differences, these experiments were conducted in colon organoids derived from three healthy individuals. *MSH4* expression was significantly increased following the CRISPR‐mediated deletion of *MLH1* and *MSH2* in organoids derived from the right colon. A nonsignificant increase in *MSH4* expression was also observed following *MSH2* editing in all three matched left colon organoids. Although we did not observe a significant increase in *MSH4* expression following CRISPR editing of all three LS‐related genes, we note that gene expression is tightly regulated by many genetic and nongenetic factors. Our confirmation of a correlation between increased *MSH4* expression and LS‐related MMR genes has important biologic implications and strongly supports a role for *MSH4* as a relevant marker of dMMR. Any causal relationship between *MSH4* expression and dMMR has yet to be determined.

The strong relationship between *MSH4* expression and dMMR also allows us to address early progression in LS. It has long been hypothesized that dMMR in LS patients was a secondary process in the development of CRC in LS.^6^, ^79^ It has been suggested that factors including the development of *Adenomatous polyposis coli* (*APC*) mutations were responsible for adenoma initiation in LS patients and that dMMR occurred only after their initiation. This was partly based on findings that LS mutations did not increase the rate of adenoma initiation but instead, accelerated the process of continued adenoma development.^79^, ^80^, ^81^ However, the belief that dMMR is only a secondary event in CRC has been challenged by the identification of dMMR crypt foci in LS patients in the absence of CRC.^6^, ^82^, ^83^ Thus, it has been proposed that dMMR may indeed initiate adenoma formation, which then either requires a “second hit” or may lead to CRC directly.^6^, ^79^ By identifying *MSH4* as a robust marker of dMMR and determining its presence in organoids derived from normal colon biopsies of LS subjects, our data support the finding of dMMR as an early event in LS patients in the absence of CRC, providing some insight into the temporal order of events for CRC initiation.

Next, we were interested in better defining a clinical role for *MSH4* gene expression across three TCGA cancer cohorts by examining the relationship between *MSH4* gene expression and TMB. We found that *MSH4* expression was significantly associated with TMB. It has been theorized that TMB leads to downstream activation of the adaptive immune response by producing increased levels of neoantigens^78^, ^84^ and maybe an important biomarker for immune checkpoint inhibition response (ICI).^85^ Conversely, individuals with low TMB (<5) have displayed poor responses to ICI.^86^ Nonsynonymous measures of TMB are somewhat challenging to define, requiring the number of somatic mutations to be quantified within a coding region of a tumor genome. That *MSH4* expression is significantly associated with TMB, implicates it as a novel biomarker for TMB, and its role in ICI should be considered. A previous report has shown that an individual with an MSH4 mutation and high TMB showed a complete response to ICI.^78^ Despite the significant associations observed between TMB and *MSH4* in all three data sets, a more complicated pattern emerged in TCGA‐UCEC, whereby individuals with POLE subtypes were more strongly associated with TMB than those with MSI‐H. Only after the removal of these individuals was the association between *MSH4* expression and TMB identified in this data set. Proofreading defects in *POLE* have been implicated in the establishment of a hypermutator phenotype but do not lead to MSI‐H phenotypes in the absence of dMMR.^78^ Given the cancer‐specific heterogeneity observed in our findings, more work is needed to better determine the relationship between the mutational landscape of a tumor, *MSH4*, and TMB. Future studies should also consider whether baseline expression of *MSH4* in more accessible samples, such as blood or saliva, may be a useful predictive marker for the success of ICI.

There are limitations to this study. For example, therapeutic drug use was not considered a covariate in our regression analysis and measures were taken from each individual at only one timepoint. We aimed to contextualize the DMRs identified between LS and healthy colon organoids within the framework of MSI‐H versus MSS tumors. However, a number of important differences exist between hereditary and nonhereditary MSI‐H tumor phenotypes. For example, the vast majority of nonhereditary MSI‐H CRC tumors occur in the right colon and are driven by hypermethylation or somatic mutation of *MLH1*. This is in contrast to LS, where as many as 45% of CRC tumors may develop outside the right colon.^77^ Nonhereditary MSI‐H tumors predominately develop along the serrated pathway, which is not true for LS tumors, which may arise from one of three distinct pathways.^79^ This may have led to some LS‐specific findings being overlooked. We compared results from organoids of LS versus healthy subjects to MSI‐H versus MSS tumors to provide validation given the initial relatively small sample size. Future, larger studies should explore the possibility that differences exist between LS and nonhereditary forms of MSI‐H tumors. Of note, some additional cancers such as bladder and breast cancers also present with MSI‐H tumors. However, the relative numbers of these tumors within the TCGA database are nominal.^12^ As such, the role of *MSH4* as a marker of dMMR in these cancers was not considered. Second, our use of CRISPR‐cas9 editing technology to delete known MMR genes implicated in LS suggested a direct relationship between *MSH4* expression and MMR status in the colon organoid model. However, we do not provide data on whether MSH4 directly alters MMR gene expression, or whether this occurs through *MSH4* DNA methylation. Third, a positive relationship between DNA methylation at *MSH4* and increased gene expression was observed. Given this surprising, positive relationship, future studies should also look to better disentangle the DNA methylation signal observed here. Most studies of DNA methylation on the Illumina EPIC array, look at the composite effects of 5‐methylcytosine (5mC) and 5‐hydroxymethylcytosine. However, the latter is present in most cell types at vastly lower levels.^87^ Through the use of oxidative bisulfite treatment, the precise role of these two epigenetic marks on gene expression of *MSH4* may be better defined. Importantly, at the gene body, differences in 5hMC have been previously associated with increased gene expression of expected targets.^87^ Further research is needed to determine whether DNA hypermethylation of the *MSH4* locus identified may serve as a relevant biomarker not only for LS and more generally, MSI‐H tumors, but also whether differences at this locus may have causal biologic and mechanistic relevance in MSI‐H tumor development. This work should also consider whether colon organoid levels of DNA hypermethylation of the identified locus or increased *MSH4* expression correlates well with other, more readily accessible tissues such as blood or buccal cells. However, these tissues were not collected for these samples as part of our organoid biorepository and are beyond the scope of this initial study. Verification of these changes in such tissues would improve the potential clinical relevance of our findings.

## 5 CONCLUSION

We have identified *MSH4* as a consistent feature in colon organoids of LS versus healthy subjects and in MSI‐H versus MSS/MSI‐L tumors across different cancers, establishing *MSH4* as a novel marker of LS and dMMR. This finding was also seen in normal LS colon organoids adding weight to the hypothesis that dMMR occurs prior to tumorigenesis in LS. Further work will be needed to determine if there is any causal relationship between *MSH4* and MSI‐H tumor development.

## AUTHOR CONTRIBUTIONS

**Matthew Devall:** Conceptualization (equal); data curation (equal); formal analysis (lead); investigation (equal); methodology (lead); resources (equal); software (lead); validation (equal); visualization (lead); writing – original draft (lead); writing – review and editing (equal). **Mourad W Ali:** Investigation (equal); methodology (equal); resources (equal); validation (equal); writing – review and editing (equal). **Stephen Eaton:** Data curation (equal); investigation (equal); methodology (equal); resources (equal); validation (equal); writing – review and editing (equal). **Daniel Weisenberger:** Investigation (equal); methodology (equal); validation (equal); writing – review and editing (equal). **Steven M Powell:** Data curation (equal); investigation (equal); resources (equal); writing – review and editing (equal). **Li Li:** Investigation (equal); methodology (equal); writing – original draft (equal); writing – review and editing (equal). **Graham Casey:** Conceptualization (equal); funding acquisition (lead); investigation (equal); methodology (equal); project administration (lead); resources (equal); supervision (lead); writing – original draft (equal); writing – review and editing (equal). Investigation (equal); writing – review and editing (equal).

## FUNDING STATEMENT

This study was supported by grants NIH R01 CA143237 and NIH R01 CA204279 (to G.C.).

## CONFLICT OF INTEREST STATEMENT

The authors declare no potential conflict of interest.

## Supporting information


Figure S1.

Figure S2.

Table S1.

Tables S2‐S8.

## Data Availability

Novel EPIC array and RNA‐seq data generated in this study have been uploaded to Gene Expression Omnibus^32^ and can be found under accession number GSE210018.
