# Supplementary figures and images for: Multi‐omic analysis in normal colon organoids highlights MSH4 as a novel marker of defective mismatch repair in Lynch syndrome and microsatellite instability
Source: Cancer Med. 2023 May 10;12(12):13551–72. doi: 10.1002/cam4.6048 (PMC10315803; doi:10.1002/cam4.6048)

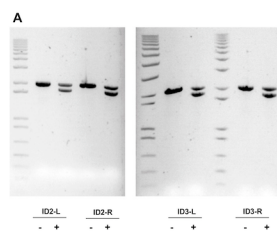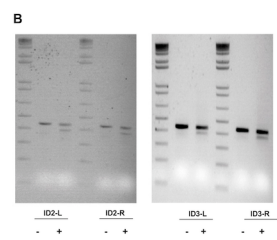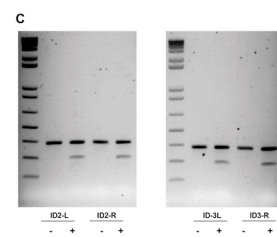

Supplement: Supplementary file 1 — Figure S1. [file CAM4-12-13551-s001.pdf]

Replicate 1 2 3

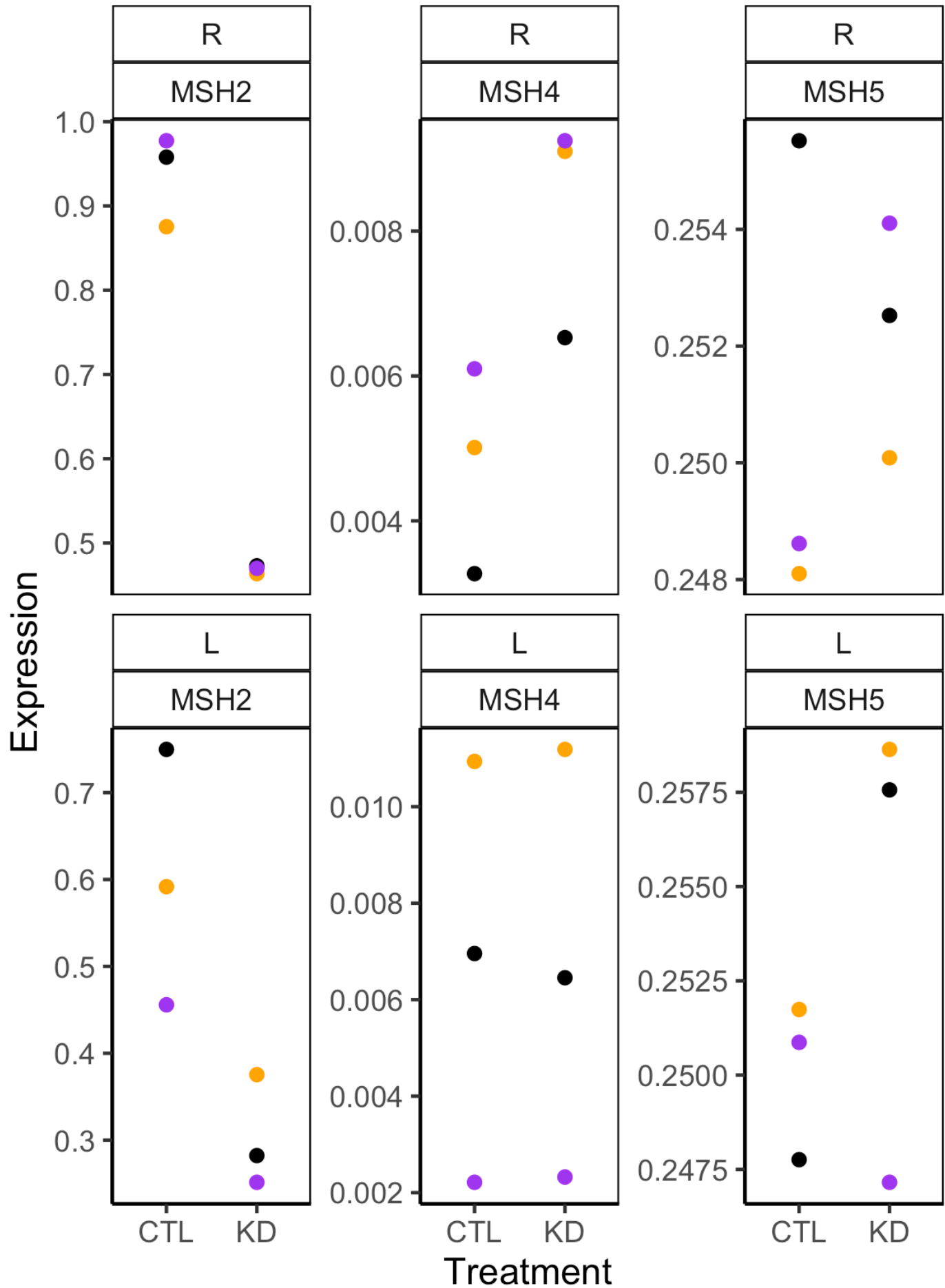

Supplement: Supplementary file 2 — Figure S2. [file CAM4-12-13551-s004.pdf]
