# Supplementary material for: Multi‐omic analysis in normal colon organoids highlights MSH4 as a novel marker of defective mismatch repair in Lynch syndrome and microsatellite instability
Source: Cancer Med. 2023 May 10;12(12):13551–72. doi: 10.1002/cam4.6048 (PMC10315803; doi:10.1002/cam4.6048)
Supplement: Supplementary file 3 — Table S1. [file CAM4-12-13551-s002.docx]

| Stage, setting | MMR (P/D)^+^ | Treatment | Study/Ref |
| --- | --- | --- | --- |
| II/III, adjuvant | P | FOLFOX (3-6 mo), or CAPEOX (3-6 mo), or 5FU/Capecitabine (6 mo) | Andre et al.(1)  Schmoll et al.(2)  Twelves et al.(3) |
| IIIC, neoadjuvant | P | FOLFOX (3-6 mo) | FOxTROT trial(4) |
| II/III, neoadjuvant | D | Dostarlimab (6 mo), or  Nivo/Ipi (6 wks) | Cercek et al.(5)  NICHE trial (6) |
| IV | P | FOLFOX +/- bevacizumab, or  CAPEOX +/- bevacizumab, or  FOLFIRI +/- bevacizumab, or  FOLFIRINOX +/- bevacizumab  Regorafenib  Trifluridine + tipiracil +/- bev | deGramont et al(7), Emmanouilides et al.(8)  Saltz et al.(9)  Andre et al(10), FIRE-3 study(11)  TRIBE trial(12)  CORRECT trial(13)  RECOURSE trial(14) |
| IV (RAS/RAF WT) | P | FOLFOX + EGFRi*, or  FOLFIRI + EGFRi, or | Venook et al.(15)  Peeters et al.(16) |
| IV (HER2 amplified, RAS/RAF WT) | P | Trastuzumab + pertuzumab, or  Trastuzumab + lapatinib, or  Trastuzumab + tucatinib  Fam-trastuzumab deruxtecan-nxki | myPathway trial(17)  HERACLES trial(18)  DESTINY-CRC01 trial(19) |
| IV (BRAF V600E) | P/D | Encorafenib + EGFRi | BEACON CRC trial(20) |
| IV | D | Pembrolizumab  Nivo/Ipi  Dostarlimab-gxly | Le et al.(21)  Overman et al.(22)  GARNET trial(23) |

**Supplemental Table 1:** A summary of the major clinical trials that have been considered for the treatment of MSI-H and MSS tumors.

+ MMR proficient (MMR-P, or MSS tumors). MMR deficient (dMMR, or MSI-H tumors).

* EGFRi: either cetuximab or panitumumab

**Supplemental References**

1. Andre T, Boni C, Mounedji-Boudiaf L, Navarro M, Tabernero J, Hickish T, et al. Oxaliplatin, fluorouracil, and leucovorin as adjuvant treatment for colon cancer. N Engl J Med. 2004;350(23):2343-51.

2. Schmoll HJ, Cartwright T, Tabernero J, Nowacki MP, Figer A, Maroun J, et al. Phase III trial of capecitabine plus oxaliplatin as adjuvant therapy for stage III colon cancer: a planned safety analysis in 1,864 patients. J Clin Oncol. 2007;25(1):102-9.

3. Twelves C, Wong A, Nowacki MP, Abt M, Burris H, 3rd, Carrato A, et al. Capecitabine as adjuvant treatment for stage III colon cancer. N Engl J Med. 2005;352(26):2696-704.

4. Morton D, Seymour M, Magill L, Handley K, Glasbey J, Glimelius B, et al. Preoperative Chemotherapy for Operable Colon Cancer: Mature Results of an International Randomized Controlled Trial. J Clin Oncol. 2023;41(8):1541-52.

5. Cercek A, Lumish M, Sinopoli J, Weiss J, Shia J, Lamendola-Essel M, et al. PD-1 Blockade in Mismatch Repair-Deficient, Locally Advanced Rectal Cancer. N Engl J Med. 2022;386(25):2363-76.

6. Chalabi M, Fanchi LF, Dijkstra KK, Van den Berg JG, Aalbers AG, Sikorska K, et al. Neoadjuvant immunotherapy leads to pathological responses in MMR-proficient and MMR-deficient early-stage colon cancers. Nat Med. 2020;26(4):566-76.

7. de Gramont A, Figer A, Seymour M, Homerin M, Hmissi A, Cassidy J, et al. Leucovorin and fluorouracil with or without oxaliplatin as first-line treatment in advanced colorectal cancer. J Clin Oncol. 2000;18(16):2938-47.

8. Emmanouilides C, Sfakiotaki G, Androulakis N, Kalbakis K, Christophylakis C, Kalykaki A, et al. Front-line bevacizumab in combination with oxaliplatin, leucovorin and 5-fluorouracil (FOLFOX) in patients with metastatic colorectal cancer: a multicenter phase II study. BMC Cancer. 2007;7:91.

9. Saltz LB, Clarke S, Diaz-Rubio E, Scheithauer W, Figer A, Wong R, et al. Bevacizumab in combination with oxaliplatin-based chemotherapy as first-line therapy in metastatic colorectal cancer: a randomized phase III study. J Clin Oncol. 2008;26(12):2013-9.

10. Andre T, Louvet C, Maindrault-Goebel F, Couteau C, Mabro M, Lotz JP, et al. CPT-11 (irinotecan) addition to bimonthly, high-dose leucovorin and bolus and continuous-infusion 5-fluorouracil (FOLFIRI) for pretreated metastatic colorectal cancer. GERCOR. Eur J Cancer. 1999;35(9):1343-7.

11. Heinemann V, von Weikersthal LF, Decker T, Kiani A, Vehling-Kaiser U, Al-Batran SE, et al. FOLFIRI plus cetuximab versus FOLFIRI plus bevacizumab as first-line treatment for patients with metastatic colorectal cancer (FIRE-3): a randomised, open-label, phase 3 trial. Lancet Oncol. 2014;15(10):1065-75.

12. Cremolini C, Loupakis F, Antoniotti C, Lupi C, Sensi E, Lonardi S, et al. FOLFOXIRI plus bevacizumab versus FOLFIRI plus bevacizumab as first-line treatment of patients with metastatic colorectal cancer: updated overall survival and molecular subgroup analyses of the open-label, phase 3 TRIBE study. Lancet Oncol. 2015;16(13):1306-15.

13. Grothey A, Van Cutsem E, Sobrero A, Siena S, Falcone A, Ychou M, et al. Regorafenib monotherapy for previously treated metastatic colorectal cancer (CORRECT): an international, multicentre, randomised, placebo-controlled, phase 3 trial. Lancet. 2013;381(9863):303-12.

14. Mayer RJ, Van Cutsem E, Falcone A, Yoshino T, Garcia-Carbonero R, Mizunuma N, et al. Randomized trial of TAS-102 for refractory metastatic colorectal cancer. N Engl J Med. 2015;372(20):1909-19.

15. Venook AP, Niedzwiecki D, Lenz HJ, Innocenti F, Fruth B, Meyerhardt JA, et al. Effect of First-Line Chemotherapy Combined With Cetuximab or Bevacizumab on Overall Survival in Patients With KRAS Wild-Type Advanced or Metastatic Colorectal Cancer: A Randomized Clinical Trial. JAMA. 2017;317(23):2392-401.

16. Peeters M, Price TJ, Cervantes A, Sobrero AF, Ducreux M, Hotko Y, et al. Randomized phase III study of panitumumab with fluorouracil, leucovorin, and irinotecan (FOLFIRI) compared with FOLFIRI alone as second-line treatment in patients with metastatic colorectal cancer. J Clin Oncol. 2010;28(31):4706-13.

17. Meric-Bernstam F, Hurwitz H, Raghav KPS, McWilliams RR, Fakih M, VanderWalde A, et al. Pertuzumab plus trastuzumab for HER2-amplified metastatic colorectal cancer (MyPathway): an updated report from a multicentre, open-label, phase 2a, multiple basket study. Lancet Oncol. 2019;20(4):518-30.

18. Sartore-Bianchi A, Trusolino L, Martino C, Bencardino K, Lonardi S, Bergamo F, et al. Dual-targeted therapy with trastuzumab and lapatinib in treatment-refractory, KRAS codon 12/13 wild-type, HER2-positive metastatic colorectal cancer (HERACLES): a proof-of-concept, multicentre, open-label, phase 2 trial. Lancet Oncol. 2016;17(6):738-46.

19. Siena S, Di Bartolomeo M, Raghav K, Masuishi T, Loupakis F, Kawakami H, et al. Trastuzumab deruxtecan (DS-8201) in patients with HER2-expressing metastatic colorectal cancer (DESTINY-CRC01): a multicentre, open-label, phase 2 trial. Lancet Oncol. 2021;22(6):779-89.

20. Kopetz S, Grothey A, Yaeger R, Van Cutsem E, Desai J, Yoshino T, et al. Encorafenib, Binimetinib, and Cetuximab in BRAF V600E-Mutated Colorectal Cancer. N Engl J Med. 2019;381(17):1632-43.

21. Le DT, Uram JN, Wang H, Bartlett BR, Kemberling H, Eyring AD, et al. PD-1 Blockade in Tumors with Mismatch-Repair Deficiency. N Engl J Med. 2015;372(26):2509-20.

22. Overman MJ, Lonardi S, Wong KYM, Lenz HJ, Gelsomino F, Aglietta M, et al. Durable Clinical Benefit With Nivolumab Plus Ipilimumab in DNA Mismatch Repair-Deficient/Microsatellite Instability-High Metastatic Colorectal Cancer. J Clin Oncol. 2018;36(8):773-9.

23. Oaknin A, Gilbert L, Tinker AV, Brown J, Mathews C, Press J, et al. Safety and antitumor activity of dostarlimab in patients with advanced or recurrent DNA mismatch repair deficient/microsatellite instability-high (dMMR/MSI-H) or proficient/stable (MMRp/MSS) endometrial cancer: interim results from GARNET-a phase I, single-arm study. J Immunother Cancer. 2022;10(1).
